# Mechanistic insights into molecular evolution of species-specific differential glycosaminoglycan binding surfaces in growth-related oncogene chemokines

**DOI:** 10.1098/rsos.171059

**Published:** 2017-09-13

**Authors:** Khushboo Gulati, Minal Jamsandekar, Krishna Mohan Poluri

**Affiliations:** 1 Department of Biotechnology, Indian Institute of Technology Roorkee, Roorkee, 247667, Uttarakhand, India; 2 Centre for Nanotechnology, Indian Institute of Technology Roorkee, Roorkee, 247667, Uttarakhand, India

**Keywords:** molecular evolution, chemokines, glycosaminoglycans, neutrophil trafficking, growth-related oncogenes

## Abstract

Chemokines are chemotactic cytokines involved in leucocyte trafficking to infected tissue. Growth-related oncogene (GRO) chemokines namely CXCL1, CXCL2 and CXCL3 are neutrophil activating chemokines sharing a conserved three-dimensional structure, but encompassing functional diversity due to gene duplication and evolutionary events. However, the evolutionary mechanisms including selection pressures involved in diversification of GRO genes have not yet been characterized. Here, we performed comprehensive evolutionary analysis of GRO genes among different mammalian species. Phylogenetic analysis illustrated a species-specific evolution pattern. Selection analysis evidenced that these genes have undergone concerted evolution. Seventeen positively selected sites were obtained, although the majority of the protein is under purifying selection. Interestingly, these positively selected sites are more concentrated on the C-terminal/glycosaminoglycan (GAG) binding and dimerization segment compared to receptor binding domain. Substitution rate analysis confirmed the C-terminal domain of GRO genes as the highest substituted segment. Further, structural analysis established that the nucleotide alterations in the GAG binding domain are the source of surface charge modulation, thus generating the differential GAG binding surfaces and multiple binding sites as per evolutionary pressure, although the helical surface is primordial for GAG binding. Indeed, such variable electrostatic surfaces are crucial to regulate chemokine gradient formation during a host's defence against pathogens and also explain the significance of chemokine promiscuity.

## 1. Introduction

**Figure 1.**
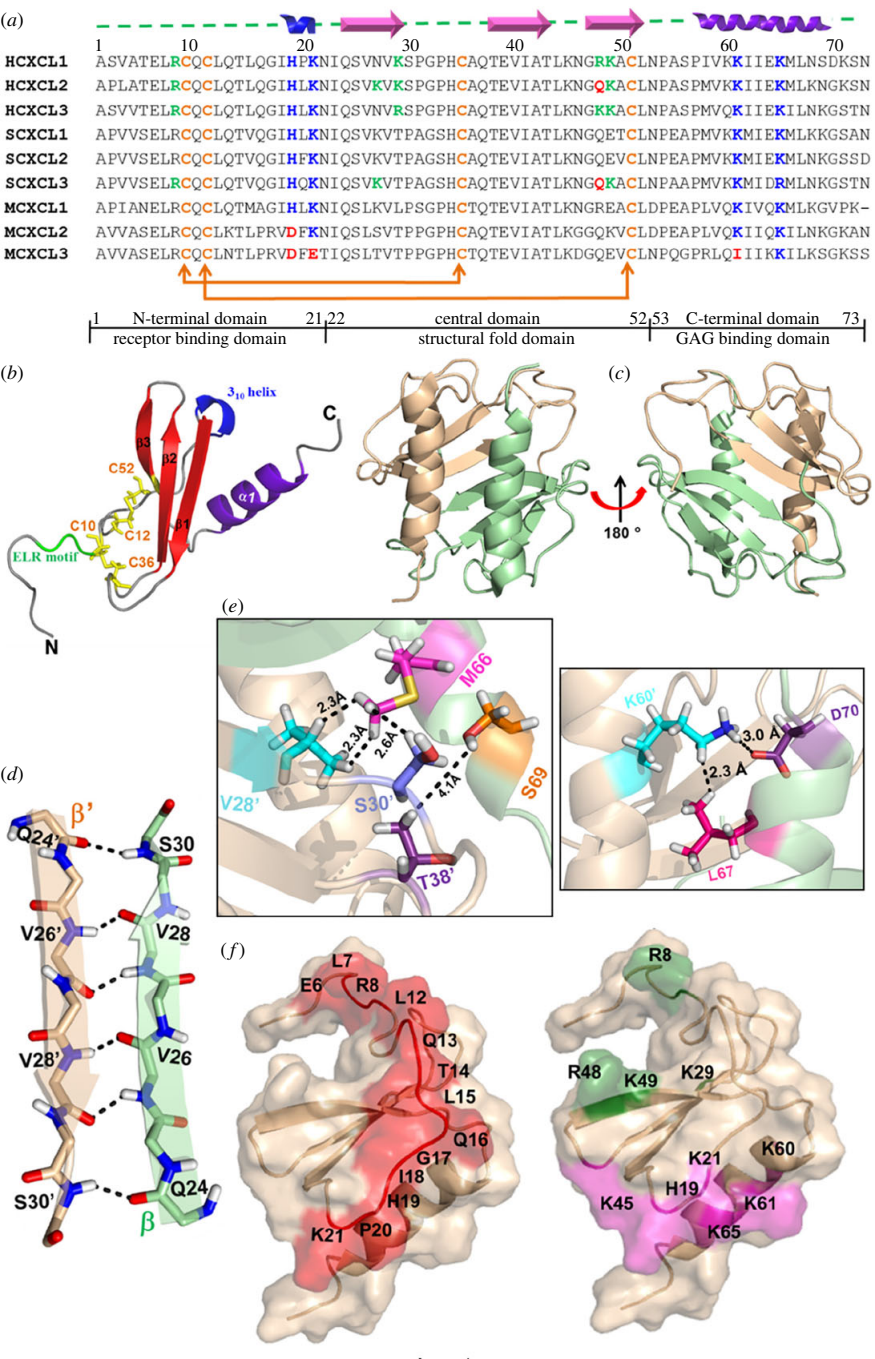
(*a*) Amino acid sequences of GRO family of chemokines from human (H), horse (S) and murine (M). Residues involved in canonical α-helical surface GAG binding are marked in blue and those specific to β-sheet surface GAG binding are marked in green; charge alteration among these residues is highlighted with red. (*b*) Structural elements in the monomeric structure of ELR-CXC chemokines. (*c*) Dimeric structure of HCXCL1. (*d*) Backbone H-bonding of β_1_–β_1_′ residues of dimer interface. (*e*) Essential dimer interface contacts between C-terminal helices and with β-strands. (*f*) Surface structure of HCXCL1 monomer marked with receptor binding residues (red) and GAG binding residues forming α-domain (pink) and β-domain (green).

Chemokines are small (8–10 kD) signalling entities involved in numerous physiological and pathological events including chemotaxis, inflammation, angiogenesis, haematopoiesis, tumourigenesis etc. [1,2]. They associate with cell surface glycosaminoglycans (GAGs) and the G-protein coupled receptors present on leucocytes to perform these biological functions [3,4]. Humans express around 50 chemokines and are segregated into four different classes (CC, CXC, CX3C, C) based on the position of N-terminal cysteine residues [5]. They share a structurally conserved monomeric fold, comprising a long disordered N-terminal domain followed by a 3_10_ helix, three anti-parallel β-strands and a C-terminal α-helix (figure 1*a*,*b*). Chemokines essentially interact with the receptors through their N-terminal domain (receptor binding domain), and with GAGs through 3_10_ helix and C-terminal helix (GAG binding domain; figure 1*a*). Chemokines can oligomerize into dimers, tetramers and other higher-order oligomers [6]. They form two types of dimers, namely CC and CXC type dimers. CXC-type dimer is globular which is formed by the antiparallel arrangement of two C-terminal α-helices on the top of six-stranded antiparallel β-sheet (figure 1*c*). The major hallmarks for CXC dimerization involve the formation of six backbone hydrogen bonds between the β_1_–β_1_′ strands of both the monomeric units (figure 1*d*). Several other intermolecular contacts for CXC dimer formation include hydrophobic, electrostatic/van der Waals interactions between the α-helices (α–α′) and with β-strand (α–β′) residues (figure 1*d*,*e*).

Evolutionary studies on chemokine family established that chemokines have originated from a single ancestor, 650 Mya, through a number of duplication events and are still progressing [5,7,8]. Studies involving the genomic arrangement of chemokines by syntenic analysis across the genomes of mammalian species have suggested that the chemokine family has expanded mainly through tandem gene duplication events [5,9–11]. Numerous other evolutionary processes such as birth and death, insertion/deletion alteration of nucleotides etc. have also contributed in shaping this multigene protein family [12–19].

Although many reports have exposed various evolutionary perspectives about these molecular emissaries, still there is a lag in tracing their structural and functional implications using evolutionary concepts. To throw light on the evolutionary characteristics of chemokines, we have chosen growth-related oncogene (GRO) chemokines belonging to the family of neutrophil activating chemokines (NACs). NACs comprise seven members with a signature ELR (Glu-Leu-Arg) motif at the N-terminus. GRO chemokines that are expressed during melanoma tumours, and are essential for wound healing activities, comprise three highly similar proteins known as GRO-α (CXCL1), GRO-β (CXCL2) and GRO-γ (CXCL3) [20–23]. Despite their super-close relativity in sequence and structure, they are involved in different functions like differential receptor activation, binding and expression patterns [24–26]. This can be attributed to their differential homo/hetero oligomerization, binding to receptors/GAGs or a combination of such molecular recognition events [24,27]. N-terminal residues including E6-R8 and L12-K21 constitute the receptor binding domain of NACs and conserved GAG binding residues (H19, K21, K45, K61, K65) are distributed on the helices (α-domain) (figure 1*f*) [28]. Further, a unique GAG binding β-domain surface comprising residues R8, R48, K49, K29 in human CXCL1 chemokine was recently reported [29] (figure 1*f*).

To delineate the evolutionary mechanisms that tune with the structural and functional aspects of GRO chemokines, we analysed GRO genes across different mammalian species using phylogenetic, selection, substitution rate analysis, conservation scores, nucleotide alterations and electrostatic surface potentials. Results of our analysis revealed that the GRO genes underwent species-specific duplication pattern, experienced positive selection extensively in the GAG binding domain compared with the receptor binding domain. Such an evolutionary regulation process resulted in chemokine structures with same tertiary folds but with varied surface potentials/oligomerization efficacies. We believe that our evolutionary analysis reported here has provided a liaison to understand the role of chemokine promiscuity in leucocyte trafficking.

## 2. Material and methods

### 2.1. Sequence alignment and phylogenetic analysis

A total of 83 gene/amino acid sequences of GRO chemokines (CXCL1-38, CXCL2-21, CXCL3-24) from different mammalian species were obtained from the sequence databases of GENBANK (http://www.ncbi.nlm.nih.gov/genbank/), Uniprot (http://www.uniprot.org/) and Ensemble (http://www.ensembl.org/), for which the unique IDs are listed in electronic supplementary material, table S1. Out of these sequences, we found all three GRO gene sequences for the following species: primates (human, chimpanzee, orangutan, gibbon, crab eating macaque), lagomorpha (rabbit), rodents (rat, murine, chinese hamster), and laurasiatheria (horse, cow). Only two gene sequences for walrus, bison, white rhinoceros, pig and buffalo of laurasiatheria were used. Although evolutionary analysis was performed by using the complete set of 83 sequences, we used the species containing either two or three GRO genes for all the analysis related to gene duplication phenomenon.

Multiple sequence alignment for the nucleotide sequences of GRO chemokines was generated using CLUSTAL Omega (https://www.ebi.ac.uk/Tools/msa/clustalo/) by employing default settings for gap extension and gap opening penalty [30]. Multiple sequence alignment thus obtained was used as input data for molecular evolutionary genetic analysis (MEGA 6) software [31]. Phylogenetic tree of GRO family chemokines among different mammalian species was constructed using neighbour joining method based on p-distance in MEGA 6 [31]. Reliability of the tree was assessed by bootstrap method using 1000 bootstrap replications that resulted in bootstrap proportion for each internal branch in the tree.

### 2.2. Selection analysis

Multiple sequence alignment of GRO nucleotide sequences was screened for the gene recombination using genetic algorithm recombination detection (GARD) approach of DATAMONKEY server [32,33]. In addition, GENECONV method of the RDP4 program was used to identify the gene conversion events [34].

Codon-based maximum-likelihood method was employed to test positive selection in GRO genes and to infer amino acid sites under positive selection during evolution using DATAMONKEY server and codeml program in the PAML 4.9a [33,35]. DATAMONKEY programs include: single likelihood ancestor counting (SLAC), fixed-effect likelihood (FEL), internal fixed effects likelihood (iFEL), mixed-effect model evolution (MEME), branch-site random effect likelihood (REL), fast unbiased Bayesian approximation (FUBAR) [33,36–38]. For SLAC, FEL, iFEL and MEME methods, a *p*-value of 0.1 was used. *p*-Value was set to 0.95 for FUBAR, and a Bayes factor of greater than 95 was used for REL. For codeml program, once the positive selection was confirmed, naive or Bayes empirical Bayes approach was used to calculate the posterior probability for all the sites evolved under positive selection [39]. As reported previously, the resulting positively selected amino acid sites that were obtained at least by two methods were considered [17,40–43]. Further, to examine the nature of duplication (concerted/divergent) among GRO genes, we compared the pairwise values of d*N* and d*S* for all the species.

### 2.3. Conservation score and substitution rates

To determine the site-specific conservation score of GRO proteins, ConSurf server was used [44]. Conservation profile for each of the sites was generated using WebLogo [45]. Nucleotide substitution rates for GRO genes were determined using p-distance model in MEGA 6.0 [31].

### 2.4. Computation of pairwise omega values among different species

To assess the variations among the non-synonymous/synonymous substitution rate ratios among different GRO domains (figure 1*a*), pairwise omega values were computed for three GRO domain sequences independently using Nei–Gojobori method with JCODA [46,47].

### 2.5. Coevolution analysis

Coevolving residues in the GRO genes were predicted using MISTIC (mutual information server to infer coevolution) server [48]. Analysis was done using the available structures of HCXCL1, HCXCL2 and MCXCL2. Average mutual information (AMI) score was calculated for all the coevolving pairs of positively selected residues.

### 2.6. Sequence and structural analysis of growth-related oncogene chemokines

The nucleotide codon versus amino acid alterations of the CXCL1-3 sequences for all the species present in the phylogenetic tree were analysed independently by aligning amino acids with nucleotide sequences.

Structural models for human CXCL3, murine CXCL3 and horse CXCL1/CXCL2/CXCL3 were generated through homology modelling based on target template alignment using Promod II in Swiss model server by employing known GRO structures as templates (electronic supplementary material, tables S2 and S3) [49]. Electrostatic surface potential maps for human, horse and murine CXCL1-3 monomeric and dimeric chemokines were generated using vacuum electrostatics of PyMOL molecular graphic system [50].

## 3. Results

### 3.1. Growth-related oncogene genes adapted species-specific evolutionary patterns

**Figure 2.**
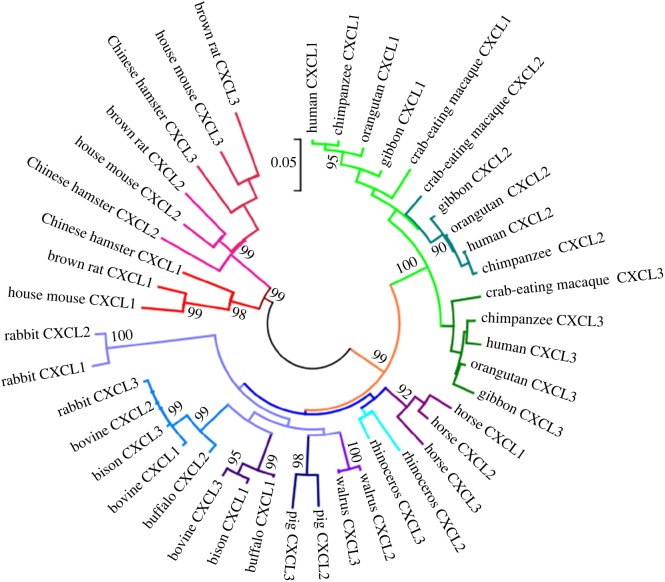
Phylogenetic tree of GRO family chemokines among different mammalian species. Scale bar represents distance that corresponds to 5% amino acid differences between the sequences.

Phylogenetic analysis exploiting the sequence data enriches us with an accurate picture of how different genes evolve, and are related to each other with respect to time and speciation. It not only provides information about the gene evolution pattern at present but also predicts the patterns of evolution that can be followed in future. To rejuvenate the historical events that have occurred during the course of evolution of GRO genes, we constructed a phylogenetic tree using the amino acid sequences of GRO proteins (figure 2). Broadly, the tree topology displayed three major clades depending on the GRO genes belonging to different suites of species, namely primates, laurasiatheria and rodents. Distribution of these nodes in the tree revealed a distant relationship among GRO genes. Moving towards the inner branches, the tree has unlocked the trends of their inherent duplication and speciation events. Within primates, CXCL1, CXCL2 and CXCL3 are more closely related to their orthologues. Similar topology was observed in rodents, implying duplication as a pre-speciation event. A contrasting trend was marked in the case of laurasiatheria, in which paralogues of GRO chemokines are more closely related than to their orthologues.

Further, to establish the origin of such varied phylogenetic features of the GRO chemokines in mammalian species, we have traced their ancestral origin and classification (electronic supplementary material, figure S1). Boroeutheria with two superorder branches, euarchontoglires and laurasiatheria, were considered to analyse the distinguishable patterns of GRO chemokine evolution. The primates, lagomorpha and rodents belong to the superorder euarchontoglires, where they show close relationship to their paralogues implicating that duplication of GRO genes in euarchontoglires is a pre-speciation event and confirms its ancestral origin. In contrast to euarchontoglires, the superorder laurasiatheria comprising perissodactyla, cetoartiodactyla and carnivora species showed more similarity among the paralogues of GRO genes implying that the duplication of GRO genes in ferreungulata is probably a post-speciation event. Identification of the ancestral origin of laurasiatheria with current set of data of GRO chemokines is not feasible. Full genome annotation of more mammalian species under laurasiatheria is required to reconsider their evolutionary patterns and to consider magnoorder Boreoeutheria as the most probable ancestor for duplicated GRO gene family.

Moving towards the other panel of the tree, one alluring difference was marked in the case of rabbit GRO genes. Despite rabbit being a species of euarchontoglires, it forms a clade with laurasiatheria. Notably, it has been observed that within rabbit GRO genes, CXCL3 shows a different behaviour and is close to bovine CXCL2 and forms a single node. In contrast, the other two paralogues of rabbit (CXCL1 and CXCL2) form a separate node. Similar kind of tree topology for rabbit has also been reported earlier [16]. In summary, this tree outlines the fact that GRO genes have followed specific evolutionary patterns, which might be the outcome of selection pressures.

### 3.2. Structural basis for observed Darwinian selection criterion in growth-related oncogene chemokines

In general, after the gene duplication, newly formed genes evolve by undergoing mutations. Some of these mutational changes can be progressed/selected, whereas some can be lost. Further, changes that occur can be synonymous or non-synonymous. Studies have reported that proteins after duplication are more prone to fixation of non-synonymous changes so that they can adopt new or modified functions [51–53]. The simplest way to find out the positive Darwinian selection in the protein coding region is to compare the non-synonymous (d*N*) and synonymous substitution rates (d*S*). The difference between the two rates is measured by the ratio omega (*ω*) equal to d*N*/d*S* which depicts the effect of amino acid selection on the encoded protein of a given gene [54]. If non-synonymous mutations are dysfunctional, purifying selection will diminish or prohibit their fixation and, therefore, d*N*/d*S* will be less than 1, but when the non-synonymous mutations are neutral, then they will be fixed at the rate similar to synonymous mutations and d*N*/d*S* will be equal to 1. Non-synonymous mutations are fixed at a rate higher than that of synonymous substitutions, with d*N*/d*S* > 1 only in the case of positive Darwinian selection, a stringent criterion for detecting positive selection [55,56].

Prior to selection analysis, GRO genes were analysed for the occurrence of any gene recombination events as these events can mislead the conclusions obtained from selection analysis. Previous work by Abrantes *et al.* reported the gene conversion events between CC chemokine receptors in various mammalian species, although they are absent in their CC chemokine ligands [57,58]. In order to analyse such gene conversion events in GRO chemokines, we performed GARD and GENECOV analysis. The results evidenced no statistically significant gene recombination and gene conversion events in GRO genes. These observations are in coherence with the recombination analysis for CC chemokine ligands of CCR5 as described above [57–59].

**Figure 3.**
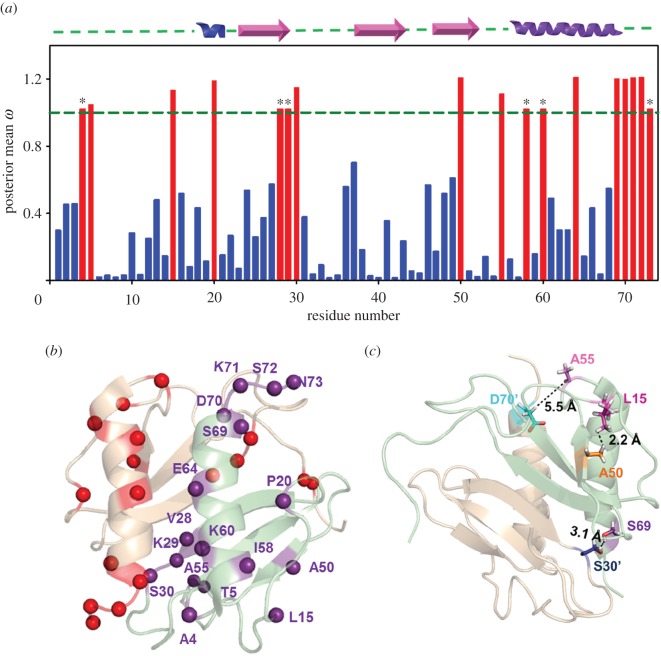
(*a*) Posterior mean *ω* at each amino acid site across the GRO genes; blue, purifying selection; red, positive selection. The horizontal line represents cutoff value for positive selection. Asterisks mark indicate the sites detected as positively selected by DATAMONKEY methods. (*b*) Positively selected residues are marked as spheres on both the monomeric units of HCXCL1 homodimer. (*c*) Significant tertiary interactions observed among the residues forming coevolved pairs are shown on HCXCL1 structure.

Selection analysis for GRO genes was performed using maximum-likelihood methods of codeml, SLAC, FEL, REL, iFEL, MEME and FUBAR. The selection analysis was individually performed for all three GRO chemokines (CXCL1, CXCL2 and CXCL3), and also for the complete set of GRO chemokines that comprises all the sets of these three duplicated genes. The main aim of performing the analysis for combined set of the GRO chemokines is to assess the correlative nature of duplication and positive selection (table 1). The datasets with individual chemokines returned 3, 5 and 3 sites for CXCL1, CXCL2 and CXCL3, respectively. Some of these sites such as A55, S72 have evolved positively in more than one GRO protein. A total of 17 positively selected sites were observed when the analysis was performed for the entire set of GRO chemokines. As expected, out of 17 sites, 9 of them are exactly the same as obtained from individual analysis. We presume that the other 8 are an outcome of the duplication phenomenon. All the selection analysis results for GRO genes are summarized in table 1 and figure 3*a*.

**Table 1.** Selection analysis of GRO chemokines using different codon-based maximum-likelihood methods. Amino acids identified by more than one method are underlined.

GRO genes	no. species	*lnL*M7	*lnL*M8	2Δ*lnL*	PAML	SLAC^a^	FEL^a^	REL^b^	iFEL^a^	MEME^a^	FUBAR^c^	total no. sites
CXCL1	37	−2994.58	−2994.24	0.54	n.a.	20, 72	20, 72	4, 15, 20, 36, 41, 45, 69, 70, 71, 72	72	20, 69, 72	20, 72	20, 69, 72
CXCL2	22	−1654	−1649.52	8.97*	5, 15, 55, 64	—	4	3, 4, 5, 13, 15, 20, 22, 26, 45, 46, 55, 62, 64, 65, 66, 68, 72, 73	5, 36	—	5, 64	4, 5, 15, 55, 64
CXCL3	24	−1913.98	−1909.55	8.85*	5, 50, 55, 72	—	50	3, 15, 22, 33, 50, 55, 58, 60, 63, 64, 65, 66, 70, 72	46, 55	50, 73	50	50, 55, 72
combined dataset	83	−5007.58	−5003.71	7.7*	5, 15, 20, 30, 50, 55, 64, 69, 70, 71, 72	4, 15, 20, 36, 70, 72	4, 15, 20, 50, 70,72	n.a.	4, 5, 46, 55,72	4, 15, 20, 28, 29, 30, 58, 60, 69, 71, 72, 73	4, 15, 20, 21, 28, 29, 30, 36, 43, 58, 60, 64, 71, 72, 73	4, 5, 15, 20, 28, 29, 30, 50, 55,58, 60, 64, 69, 70, 71, 72, 73

^a^Amino acids with significance values less than 0.1.

^b^Amino acids with Bayes factor greater than 95.

^c^Amino acids with significance values greater than or equal to 0.9.

*p < 0.05.

We then analysed the positioning and the importance of these residues with respect to the GRO chemokine structure and function. Among the positively selected residues, four of them (A4, T5, L15, P20) are in the N-terminal domain, four of them (V28, K29, S30, 50A) are in central-core domain and nine of them (A55, I58, K60, E64, S69, D70, K71, S72, N73) are in the C-terminal domain (figure 3*a*,*b*). It is evident from the data that most of the positively selected sites (10 out of 17 residues) are in the GAG binding surface (C-terminal helix + 3_10_ helix). Interestingly, these residues are mostly spanning on either sides/close proximity of experimentally demonstrated conserved GAG residues (H19, K21 on 3_10_ helix and K61, K65 on C-terminal helix) [28,29]. It has also been observed that the N-terminal region which is crucial for receptor binding and activation is under purifying selection, except the two positively selected residues (L15 and P20). Of these two residues, P20 also overlaps with GAG binding surface. These results indicate that receptor binding domain is mainly showing the signatures of purifying selection, and thus conserved among the GRO chemokines. Indeed such behaviour is very much anticipated for the receptor binding domain considering functional constraints of GRO chemokine binding to its conservative CXCR2 receptor. In contrast, the GAG binding domains have evidenced for large number of positive selection sites indicating the evolution of differential GAG binding features/GAG surfaces among GRO chemokines in different species. These results suggest that the two variable and complementary functional segments (receptor binding domain and GAG binding domain) of the GRO proteins have a selection bias in order to balance their structure–function relationship. The other interesting segment of GRO chemokines that showed positive selection is the dimer interface (V28, K29, S30) of the central-core domain. Indeed, such a specific site/segmental selection of GRO chemokines at the GAG binding and their extensions to dimer interface regions can regulate the functional activity of these chemokines by providing conformational adjustments at dimer interface, and by altering the binding energetics through modulation of positive charge potential on the surface via amino acid substitutions/gene mutations (discussed in later sections).

We further analysed the relationship among these positively selected residues in order to assess their role in coevolution. In coevolution/compensatory evolution, when one amino acid evolved at a particular position, the other amino acid evolved at different position simultaneously as a coevolving pair. We analysed the coevolving nature of all the positively selected residues (table 2, figure 3*c*). Our results demonstrated that several of these amino acids are coevolved and contribute to the structural stability through side chain networks via different tertiary interactions. For example, coevolving pair L15–A50 is involved in hydrophobic interaction. Similarly, L55–D70′ and S69–S30′ contribute to the inter-subunit interactions (figure 3*c*). It is worth noting that sometimes coevolved residues are a part of the larger interacting networks. In such cases, a direct interaction with a particular site (positively selected partner) is not evident from distance measurements as noticed for T5. Considering the conserved nature of the receptor domain, and interesting evolutionary patterns of GAG binding domain of GRO chemokines, the evolutionary analysis and discussion is more oriented towards understanding the species-specific differential GAG binding characteristics.

**Table 2.** Calculated AMI scores for the coevolving positively selected sites. Number of asterisks represents the significance of the coevolving interaction.

	coevolving amino acid pairs	AMI score	
	T5–S30	9.43 (**)	
	T5–A55	7.29 (*)	
	S30–P20	6.91 (*)	
	S30–K29	10.36 (***)	
	S30–A55	11.56 (***)	
	S69–D70	9.24 (**)	
	S69–K71	10.35 (***)	
	S69–S72	10.91 (***)	
	K71–K72	10.4 (***)	

### 3.3. Duplication of growth-related oncogene genes followed concerted evolution

Different models have been proposed for the evolution of multigene families that includes divergent evolution, concerted evolution and birth and death evolution. In divergent evolution, genes obtained after being duplicated from their parental genes acquire new gene functions independently. Whereas in concerted evolution, all the members of a gene family evolve in a concerted/coincidental manner. That is, the mutation occurring in a repeat will spread into the entire members of the gene family by the repeated occurrence of unequal crossover or gene conversion, thus resulting in homogenization of DNA sequences of all the member genes [60,61]. As unequal crossing over or gene conversions are supposed to be driving forces for concerted evolution, d*S* should be similar to or slightly higher than d*N*, irrespective of purifying selection. Moreover, if birth-and-death evolution is acting on a gene, then the rate of silent mutation should be extensive, leading d*S* to be many folds higher than d*N* [62].

To identify the evolutionary method of GRO genes, we compared the pairwise d*S* and d*N* values of GRO genes for different species. It was found that in each species the d*S* values were similar to or slightly higher than d*N* values indicating the concerted mode of evolution (table 3). Indeed, concerted evolution has been followed by many genes including ribosomal RNA genes and histone genes that code for the large quantities of proteins with similar functions and are crucial for the endurance of an organism [63,64].

**Table 3.** Pairwise values of d*S* (below diagonal) and d*N* (above diagonal) observed between GRO sequences (CXCL1/CXCL2/CXCL3 denoted as 1/2/3) from H, human; O, orangutan; S, horse; B, cow; M, mouse; R, rat.

	H1	H2	H3	O1	O2	O3	S1	S2	S3	B1	B2	B3	M1	M2	M3	R1	R2	R3
H1		0.06	0.09	0.01	0.05	0.08	0.16	0.16	0.15	0.16	0.16	0.14	0.22	0.20	0.26	0.19	0.25	0.29
H2	0.08		0.08	0.06	0.01	0.07	0.11	0.12	0.12	0.13	0.13	0.11	0.21	0.19	0.23	0.18	0.22	0.27
H3	0.15	0.15		0.09	0.08	0.01	0.12	0.12	0.11	0.12	0.12	0.10	0.24	0.18	0.27	0.21	0.21	0.29
O1	0.04	0.08	0.14		0.05	0.08	0.16	0.16	0.15	0.14	0.14	0.14	0.24	0.20	0.25	0.21	0.24	0.28
O2	0.08	0.02	0.06	0.08		0.06	0.12	0.13	0.13	0.13	0.13	0.12	0.21	0.18	0.23	0.19	0.22	0.28
O3	0.16	0.10	0.06	0.11	0.08		0.11	0.12	0.10	0.11	0.11	0.09	0.23	0.17	0.26	0.20	0.21	0.28
S1	0.28	0.41	0.41	0.29	0.35	0.34		0.04	0.05	0.11	0.11	0.07	0.20	0.20	0.25	0.17	0.21	0.26
S2	0.26	0.32	0.32	0.26	0.23	0.26	0.06		0.06	0.10	0.10	0.08	0.22	0.18	0.24	0.17	0.19	0.24
S3	0.33	0.42	0.42	0.27	0.30	0.36	0.14	0.12		0.10	0.10	0.08	0.24	0.22	0.30	0.21	0.24	0.31
B1	0.46	0.39	0.46	0.43	0.36	0.46	0.36	0.28	0.40		0.00	0.07	0.26	0.21	0.28	0.22	0.23	0.28
B2	0.42	0.36	0.42	0.39	0.33	0.42	0.33	0.25	0.36	0.02		0.07	0.26	0.21	0.28	0.22	0.23	0.28
B3	0.51	0.48	0.55	0.56	0.48	0.55	0.38	0.29	0.47	0.33	0.30		0.20	0.19	0.25	0.17	0.20	0.27
M1	0.52	0.53	0.64	0.55	0.53	0.56	0.54	0.39	0.48	0.49	0.52	0.59		0.25	0.29	0.04	0.25	0.27
M2	0.59	0.61	0.70	0.64	0.57	0.61	0.59	0.51	0.53	0.60	0.65	0.72	0.40		0.13	0.22	0.06	0.15
M3	0.70	0.69	0.67	0.66	0.68	0.59	0.54	0.55	0.45	0.42	0.56	0.72	0.51	0.24		0.24	0.13	0.09
R1	0.51	0.62	0.56	0.54	0.57	0.49	0.54	0.43	0.52	0.70	0.65	0.72	0.20	0.46	0.58		0.22	0.29
R2	0.72	0.67	0.79	0.78	0.65	0.69	0.63	0.55	0.65	0.64	0.69	0.71	0.52	0.23	0.24	0.53		0.16
R3	0.96	0.88	0.90	1.04	0.68	0.79	0.65	0.66	0.71	0.76	0.82	0.84	0.54	0.36	0.16	0.68	0.20	

### 3.4. Substitution rate analysis of growth-related oncogene proteins

**Figure 4.**
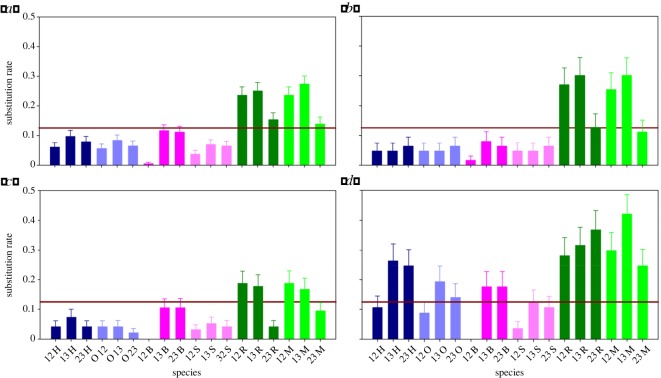
Substitution rates for (*a*) complete GRO sequences, and individual domains, namely: (*b*) N-terminal, (*c*) central and (*d*) C-terminal domain. H, human; O, orangutan; S, horse; B, cow; R, rat; M, mouse; 1/2/3 represent CXCL1/CXCL2/CXCL3. The brown horizontal line represents the lower threshold of substitution rate.

Accumulation of mutations in the coding sequences of the duplicated genes greatly influences their functions. They include differential expression patterns, and various interactions with biomolecular binding partners. Our selection analysis along with the calculated amino acid conservation scores suggested that all the positively selected sites exhibit a conservation score below 70% and several of the nucleotides/amino acids that correspond to the purifying/neutral selection do vary considerably (electronic supplementary material, figure S2). Therefore, in order to throw light on the rate and nature of nucleotide substitutions resulting after duplication events, we calculated the substitution rates (figure 4). It was observed that rodent genes are more prone to substitution in comparison to other species, when substitution rates were calculated for the full-length sequence of all the species (figure 4*a*). Further, to determine the segments of the protein that have major contribution to the high substitution rates, the protein was divided into three domains: N-terminal domain, central domain and C-terminal domain (figure 1*a*). Substitution rates were calculated for each of the domains among all the species. It was observed that in primates and laurasiatherians, C-terminal domain alone showed high substitution rates, suggesting that the rest of the protein is under stringent purifying selection (figure 4*b*,*c*). However, in contrast to the higher-order mammals, both N-terminal and C-terminal domains showed high substitution rates in the rodents. Moreover, the C-terminal domain has highest propensity of substitution changes compared with the N-terminal counterpart (figure 4*d*). Further, the comparative analysis of the substitution rates of alone C-terminal domain of the rodents with that of the same in primates and laurasiatherians suggested that rodents accumulated higher substitution rates in the C-terminal domain.

**Figure 5.**
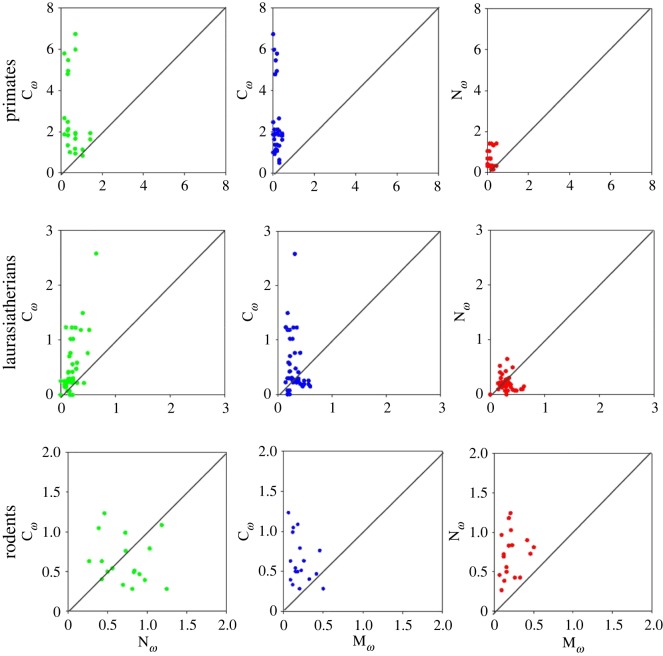
Pairwise comparison of omega values for different domains of GRO genes in different families. C*ω*, M*ω* and N*ω* represent the *ω* values for C-terminal, central and N-terminal domains, respectively.

To further support our conclusions on the variable substitution pattern, we compared the pairwise values of *ω* for different parts of the GRO proteins among different species (figure 5). In primates, higher *ω* values were observed in the case of C-terminus when compared with central and N-terminus (C-terminal domain > N-terminal domain > central domain). In laurasiatherians, a slightly different trend in *ω* values was observed, i.e. C-terminal domain > N-terminal domain = central domain. Rodents showed a distinctive pattern of *ω* values in which C-terminal domain = N-terminal domain > central domain. All these results clearly point towards the species-specific evolutionary changes regulated by variable selection pressures on different parts of the protein for a functional significance/advantage.

### 3.5. Sequence and surface properties of growth-related oncogene proteins

**Figure 6.**
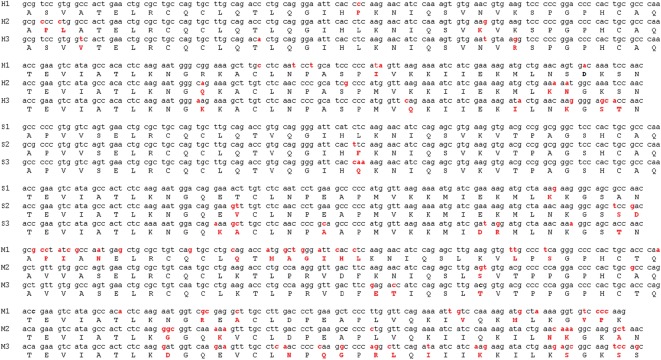
Comparative nucleotide/amino acid analysis of GRO chemokines; H, human; S, horse; M, murine; 1/2/3 represent CXCL1/CXCL2/CXCL3. Alterations in the nucleotide/amino acid sequences are highlighted in red.

Selection pressure analysis at domain level of GRO proteins prompted us to further delineate the molecular level details and sources of differential *ω* value patterns across different species. In order to dig out the differential evolution characteristics of GRO proteins across the species, we simultaneously analysed the variations in their sequences at both nucleotide and amino acid level (figure 6). We observed very small number and specifically regulated nucleotide and amino acid changes across GRO chemokines in primates and laurasiatherians. Moreover, the changes in these species are mainly through facile single-nucleotide alterations, in order to guide the duplicated sibling for a functional advantage via minimal perturbation. In contrast to this, rodent genes exhibited large number of changes with multitude of nucleotide alterations accompanying amino acid makeovers. It is worth noting that in the case of primates, the amino acid alterations are on the helical surface in GRO genes, i.e. essentially confined within the C-terminal α-helix. Whereas in rodents, apart from the N-terminal, these changes have spanned across both 3_10_ helix along with C-terminal α-helix that constitute the GAG binding surface of their positively charged residues. Moreover, the observed gene mutation(s) in the GAG binding surfaces specifically corresponds to the change(s) in amino acids that result in the charge reversal/charge neutralization of protein surface. These observations indicate that promiscuity of GRO genes under evolutionary pressure is focused towards modulating the GAG binding surfaces via minimal gene alterations through charge distribution.

**Figure 7.**
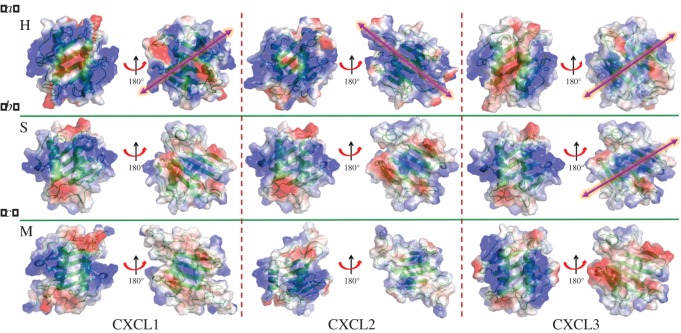
Electrostatic surface potential maps for the dimeric GRO proteins. (*a*–*c*) H, human; S, horse; M, murine chemokines, respectively. Both α-helical and β-sheet surfaces are presented. Pink arrows represent the plausible mode of GAG binding on β-sheet surfaces.

To delineate the surface architectures and electrostatic potentials that can plausibly contribute to differential GAG binding, we modelled the dimeric structures of murine, horse and human GRO chemokines (electronic supplementary material, tables S2, S3 and figure S3), and analysed their electrostatic potential surfaces (figure 7). These structural models furnished a pictorial view of distinct GAG binding surfaces that are generated by virtue of amino acid changes. Analysis on human GRO proteins (as dimers) yielded complex electrostatic surfaces and evoked the feasibility of multiple modes of GAG binding (figure 7*a*, H). It is evident from the electrostatic potential pattern that the human GRO genes will have an alternate GAG binding surface comprising the positive residues (R8, K29, R48, K49; according to HCXCL1) (figure 1*a*). In line with our evolutionary theory, a recent study on HCXCL1 confirmed the presence of two non-overlapping GAG-binding domains in HCXCL1 [29]. Interestingly, due to surface charge alterations, HCXCL2 has evolved with a contrasting 90^o^ rotated positive surface on the β-sheet/dimer interface when compared with HCXCL1 and HCXCL3 (figure 7*a*, H). Such a surface of HCXCL3 is due to the presence of K27 along with R/K29 at the dimer interface and lack of R48 at the start of β3-strand and is a resultant of single-nucleotide alteration at these two sites on the β-surface of human GRO proteins. Moreover, these changes resulted in specific positioning and shuttling of the residues within Arg and Lys (K29, R48 in hCXCL1 versus R29, K48 in HCXCL3) to fine tune their GAG binding interactions/affinities. Such a GAG binding interface at the β-domain surface has also been reported for homeostatic chemokine CXCL12 [65].

Similar analysis with horse proteins yielded surfaces that are different from the primates. In horse CXCL1 (SCXCL1), the positive surface is confined at the 3_10_ helices on the helical side with a small positive patch at the β surface of dimer interface (figure 7*b*, S). The positive charges on both the helical and the β surfaces are enhanced considerably in SCXCL3 when compared with SCXCL1 and SCXCL2 (figure 7*b*, S). This can be attributed to the single-nucleotide alterations leading to (a) charge reversal by the change of E49 to K49, (b) charge neutralization by the change of E55 to A55, and (c) contribution of enhanced positive charge with a replacement of K65 to R65 in SCXCL3. Such an enhanced positive electrostatic potential on both surfaces indicates the plausible multiple GAG binding modes of SCXCL3 when compared with SCXCL1/2.

In the case of rodents, the mechanism of differential GAG surface formation is different from those of the primates and laurasiatherians. In MCXCL1, 3_10_ helix possesses positive charge due to the presence of positively charged residues H19 and K21 (figure 7*c*, M; electronic supplementary material, figure S4). Whereas in MCXCL2, H20 is replaced by negatively charged D19, and in MCXCL3, whole positive charge at the 3_10_ helix is transformed to negative charge as a consequence of D19 and E21 (figure 7*c*, M). Such site-specific charge reversal features have resulted in varied positive surfaces in the murine CXCL1-3, although no positive potentials were observed on the β surfaces of these proteins (figure 7*c*, M). These positive surface potentials constitute single GAG binding motif on the helical surface in murine GRO proteins, and indeed previous NMR experiments on MCXCL1-trapped dimer demonstrated that GAG binds orthogonal to the inter-helical axis and the residues H20, K22 on 3_10_ helix and K60 and K64 on the C-terminal helix are crucial for GAG binding [28].

Considering all the above observed electrostatic surface variations on the mammalian GRO proteins (figures 6 and 7), we summarize that the β-sheet binding surface and its relative surface orientation(s) are confined to the primates and laurasiatherians and no such surface is evident in the rodents. Further, our analysis suggested that the electrostatic surfaces have evolved specifically among the GRO genes in a given organism, in order to serve the needs of particular species as per the evolutionary pressure, although the helical surface of GAG binding resembles the primordial mode of GAG binding in all the species. Indeed, our selection analysis supported this theme as the residues H19, K21 on 3_10_ helix and K61 and K65 on the C-terminal helix that are crucial for GAG binding are on a tight purifying selection for all the species.

## 4. Discussion

### 4.1. Mechanistic insights into evolutionary characteristics of growth-related oncogene chemokines

Immunity and defence-related genes evolve rapidly under positive selection pressures accumulating amino acid changes more rapidly than other genes. Chemokines listed as one of the 8 most rapidly changing proteins and domains [8,12]. The reason for continuous evolvability of inflammatory chemokines is intimately related to their functionality in host defence processes, as viruses copy the host endogenous chemokines, and target the host machinery. In order to fight against such evolving pathogens, the host genes need to undergo smart changes and also expand themselves to maintain their diversity.

This piece of work is an effort to furnish mechanistic insights into the evolutionary characteristics of GRO chemokines. The phylogenetic analysis provided us an overview of the fact that GRO genes have evolved in species-specific patterns. The observed phylogenetic profile for GRO genes is very much consistent with the earlier literature available on CXC chemokines [7,11,16]. Indeed, such an interesting species-specific evolution of GRO genes is an outcome of the mechanism of duplication events that has been precisely reflected in the genomic arrangements of different species including primates, laurasiatherians and rodents [11]. In rodents, all GRO proteins are present in the same transcriptional orientation in contrast to primates where CXCL2 and CXCL3 are placed in an opposite direction to CXCL1, as primate GRO gene evolution is an outcome of inverse duplication when compared with other species [10]. Such a gene positioning via duplication and species-specific nucleotide alterations under evolutionary pressure confer multiple layers of regulation to chemokine functions in terms of differential transcriptional regulation, different cellular territories of distribution, homo/hetero dimerization and receptor/GAG recognition to form cell/chemokine-specific chemotactic gradients/cell signalling events. Indeed, experimental reports on human CXCL1, CXCL2 and CXCL3 suggested that the expression of these three highly similar genes is differentially regulated in a cell- and signal-specific manner and bind to CXC receptors with varied affinities [16,24,25].

Site-specific selection analysis of duplicated GRO chemokines confirmed that the genes underwent mainly purifying/negative selection with few positively selected sites, several of them coevolving and the majority of them lying in the GAG binding domain/dimerization region. In general, the majority of the genes evolve under negative selection. For example, fork head gene family has evolved as a result of gene duplication, rapid differentiation and subsequent fixation of amino acid changes through negative selection [66]. Similarly, the post duplication charge evolution of surface charge in phosphoglucose isomerase was not driven by strong selection on individual amino acid sites but by the weak selection on large number of amino acid sites and consequently by steady directional/purifying selection on overall structural properties of the protein, which are derived from many modifiable sites [67]. In the case of GRO genes also, the modification of the electrostatic surfaces is not in line with the principles of strong positive selection. They evolved mainly under purifying selection with an exception of C-terminal domain. Such domain-specific evolutionary patterns are seen in immune signalling protein TRAF3 interacting protein 2. In this protein, the N-terminal domain that is more disordered has been subjected to positive selection, and a purifying selection was observed for the C-terminal domain which is contributing to the core structure of the protein [68]. For GRO chemokines, the selection, substitution rates and nucleotide comparison studies established that significant degree of positive selection/alterations are concentrated in the C-terminal/GAG binding domain including residues involved in the dimerization contacts. However, no positive selection has been noticed in the structural part of the protein suggesting the conserved nature of the structural fold for its chemotactic functioning.

Our analysis also established that such evolutionary changes resulted in multiple species-specific pathways for differential GAG binding such as regulated electrostatic surface in the 3_10_ helix in rodents and generation of novel β-sheet surface in primates and some of the GRO proteins in laurasiatherians, although the helical surface binding appears to be the primordial mode of GAG binding for GRO chemokines. Evolutionary analysis on formyl peptide receptors across mammals evidenced similar sort of differential electrostatic surfaces through amino acid alterations [69]. Essentially, chemokine immobilization through GAGs facilitates the formation of haptotactic gradient, and adds another layer of specificity and control to the cell migration beyond the receptor. These evolved species-specific and gene-specific GRO proteins can also have differential half-life and susceptibility to protease degradation upon GAG binding.

Further, these species-specific evolutionary events are also credited for gene inactivation and partial/complete deletions leading to pseudogenes (non-functional), like genes (novel functions) and extinct genes (erased) [9,12,13,18,70]. Like genes for CXCL1 and CXCL3 (CXCL1 L/CXCL3 L) were reported in *Macaca fascicularis* [18]. In both cases, although the contributing GAG residues are similar, the C-terminal region that is crucial for monomer–dimer equilibrium is truncated thus contributing to differential populations of GAG induced dimerization/oligomerization. On a similar note, a pseudogene (CXCL1p) found in human and chimpanzee in the inversely duplicated segment of chromosome 4, which is yet to be processed completely for complete functionality. Currently CXCL1p gene contains only two exons with one intron between, accompanied by downstream deletion resulting in a sequence that is devoid of C-terminal helix [18]. Our sequence analysis identified that in CXCL1p, residue R9 that is essential for GAG/receptor binding has been mutated to P9 thus switching off the functionality of this gene at this stage (data not shown).

Such gene alterations are not only confined to the GRO family chemokines; several other CXC chemokines also experienced these evolutionary pressures for functional advantages. For example, CXCL4L1, a homologue of platelet factor 4 (CXCL4) is a resultant of duplication of CXCL4 gene present in humans and chimpanzees, which differs in only three amino acids (P58 L, K66E and L67H) when compared with CXCL4 and exhibits potent anti-angiogenic and anti-tumourous properties. Recent structural studies have shown that C-terminal helix of CXCL4L1 adopts an open conformation due to L67H mutation. These amino acid changes coupled with the conformational transition in the C-terminal helix are responsible for the lowering of GAG binding affinities of CXCL4L1 when compared with CXCL4 [71].

Another essential feature is that, during the course of evolution, these genes have also acquired variations due to point mutations. Different types of polymorphisms like single-nucleotide polymorphisms, insertion and deletions of specific nucleotides, alternating splicing that may be associated with several diseases or sometimes may be beneficial [12]. CXCL2 which is an essential mediator in lung protection but polymorphism of a short tandem repeat (AC)n at -665 position in the promoter region altered the promoter's activity, consequently heightened the expression of CXCL2 thus contributing to severe sepsis [14]. In such pathological cases, redundancy/promiscuity of these duplicated inflammatory chemokines play a key role due to an overlap in major functions carried out by them; so that any defect in one chemokine can be easily resolved by an alternative family member to safeguard the cell from immune insults.

## 5. Concluding remarks

In summary, we reconstructed the evolutionary history of GRO genes across the diverse range of mammals. Our molecular evolutionary studies on GRO proteins threw light on the underlying principles responsible for their variable evolutionary patterns, positive selection of the protein segments, substitution rates and their lineage to GAG binding properties thus bridging our structural awareness with evolutionary programming and functional variance. Such knowledge is applicable to all the protein/chemokine families that demand a detailed lineage of structure–function relationships. Future comparative experimental studies involving protein–GAG interactions of GRO proteins from multiple species are imperative to decipher the regulatory role of multiple GAG binding surfaces/orientations during chemotactic gradients and hence neutrophil trafficking.

## Supplementary Material

GRO protein gene and structural information

## Data accessibility

The datasets supporting this article have been uploaded as part of the electronic supplementary material including accession IDs of the nucleotide sequences of all the species used in the analysis.
