# Supplementary material for: Mechanistic insights into molecular evolution of species-specific differential glycosaminoglycan binding surfaces in growth-related oncogene chemokines
Source: R Soc Open Sci. 2017 Sep 13;4(9):171059. doi: 10.1098/rsos.171059 (PMC5627130; doi:10.1098/rsos.171059)
Supplement: GRO protein gene and structural information [file rsos171059supp1.pdf]

Supplementary Material

Mechanistic Insights into Molecular Evolution of Species Specific Differential Glycosaminoglycan Binding Surfaces in GRO Chemokines

Khushboo Gulati[1], Minal Jamsandekar[1], and Krishna Mohan Poluri[1,2*]
[1]Department of Biotechnology, [2]Centre for Nanotechnology
Indian Institute of Technology Roorkee
Roorkee – 247667, Uttarakhand, India
E-mail: krishfbt@iitr.ac.in / mohanpmk@gmail.com

Stab.1: Nucleotide sequence IDs of GRO family chemokines (CXCL1, CXCL2, and CXCL3) from different mammalian species.

Species	CXCL1	CXCL2	CXCL3
African bush elephant	-	-	XM_010594141.1
Bactrian camel	XM_010969408.1		
Baiji	XM_007463218.1	XM_007463219.1	-
Bison	XM_010838198.1	-	XM_010838177.1
Black-capped squirrel monkey	XM_010342726.1	XM_010342728.1	-
Bovine	NM_175700.1	NM_001048165.1	NM_001046513.2
Brown rat	NM_030845.1	NM_053647.1	NM_138522.1
Cape golden mole	XM_006871594.1	-	-
Cat	XM_011279689.1	-	-
Cape elephant shrew	XM_006898468.1	-	-
Chinese hamster	NM_001244044.1	XM_007630617.1	XM_001244139.1
Chimpanzee	XM_001156094.4	XM_001155614.4	XM_517228.4
Chinese tree shrew	XM_006142920.2	-	-
Crab-eating macaque	AB262775.1	AB262776.2	AB262777.1
Common bottlenose dolphin	XM_004319600.1	-	-
Deer mouse	-	XM_006993515.2	-
Ferret	XM_004766349.2		
Gibbon	XM_012499279.1	ENSNLET00000010136	XM_003265742.2
Giant panda	XM_002919144.2	-	-
Gorilla	XM_004038813.1	XM_004038819.1	-
Goat	XM_013964688.1		
Golden hamster	-	XM_005068086.2	-
Guinea pig	NM_001172938.1	-	-
Gray short-tailed opossum	XM_007495669.2	-	-
Hedgehog	-	-	XM_004703375.1
House mouse	NM_008176.3	NM_009140.2	NM_203320.3
Human	NM_001511.3	NM_002089.3	NM_002090.2
Horse	NM_001309480.1	NM_001143955.1	NM_001143793.2
Killer whale	XM_012538162.1	-	-
Little brown bat	-	-	XM_006094377.2
Marmoset	XM_002745753.3	-	-
Mouflon	XM_012135521.2	-	-
Minke whale	XM_007179836.1	-	-
Nine-banded armadillo	-	-	XM_004465324.2

Naked mole rat	XM_004833919.1	-	-
Orangutan	XM_002814861.3	XM_002814867.3	XM_002814865.2
Ord's kangaroo rat	XM_013020161.1	-	-
Philipine tarsier	XM_008059500.1	-	-
Pig	NM_001001861.2	NM_001001861.2	XM_005666754.2
Platypus	XM_007669225.1	-	-
Rabbit	U95808.1	ENSOCUT00000031529	U12310.1
Rhesus macaque	NM_001032878.1	- -	NM_001032879.1
Sperm whale	XM_007126262.1	-	-
Star nosed mole	-	-	XM_004681217.2
Thirteen-lined GROund squirrel	-	XM_005333294.2	XM_005333245.1
Tasmanian devils	XM_012551956.1	-	-
Water buffalo	XM_006059633.1	XM_006059634.1	-
Walrus	-	XM_004392914.1	XM_004392941.1
West Indian manatee	-	-	XM_004383312.1
White rhinoceros	-	XM_004419171.2	XM_004419172.2
Yak	-	-	XM_005891250.2

Stab.2: PDB-ID's of all the available murine and human GRO Protein structures.

**Gene**	**PDB ID**	**Solution/ Crystal structure**	**Reference**
Human CXCL1	1MGS	Solution structure	[1]
Human CXCL1	1MSG	Solution structure	[2]
Human CXCL2	1QNK	Solution structure	[3]
MurineCXCL1	---	Modeled structure	[4]
Murine CXCL2	1MI2	Solution structure	[5]
Murine CXCL2	3N52	Crystal structure	[6]

Stab.3: Ramachandran plot statistics for modeled murine/human/horse CXCL (GRO) chemokine structures.

	Murine CXCL1		Murine CXCL3		Human CXCL3	
Residues in most favoured regions [A,B,L]	108	88.5%	107	93.9%	93	78.8%
Residues in additional allowed regions [a,b,l,p]	13	10.7%	6	5.3%	25	21.2%
Residues in generously allowed regions [~a,~b,~l,~p]	1	0.8%	1	0.9%	0	0.0%
Residues in disallowed regions	0	0.0%	0	0.0%	0	0.0%
		----------		----------		----------
Number of non-glycine and non-proline residues	122	100.0%	114	100.0%	118	100.0%
Number of end-residues (excl. Gly and Pro)	2		4		4	
Number of glycine residues (shown as triangles)	10		8		8	
Number of proline residues	12		12		8	
	----		----		----	
Total number of residues	146		138		138	

	Horse CXCL1		Horse CXCL2		Horse CXCL3	
Residues in most favoured regions [A,B,L]	110	92.45%	110	92.4%	110	92.4%
Residues in additional allowed regions [a,b,l,p]	8	6.7%	8	6.7%	8	67.6%
Residues in generously allowed regions [~a,~b,~l,~p]	1	0.8%	1	0.8%	1	0.8%
Residues in disallowed regions	0	0.0%	0	0.0%	0	0.0%
		----------		----------		----------
Number of non-glycine and non-proline residues	119	100.0%	119	100.0%	119	100.0%
Number of end-residues (excl. Gly and Pro)	3		3		3	
Number of glycine residues (shown as triangles)	8		8		8	
Number of proline residues	7		7		7	
	----		----		----	
Total number of residues	137		137		138	

Sfig.1: Schematic showing a brief evolutionary summary of mammalian species

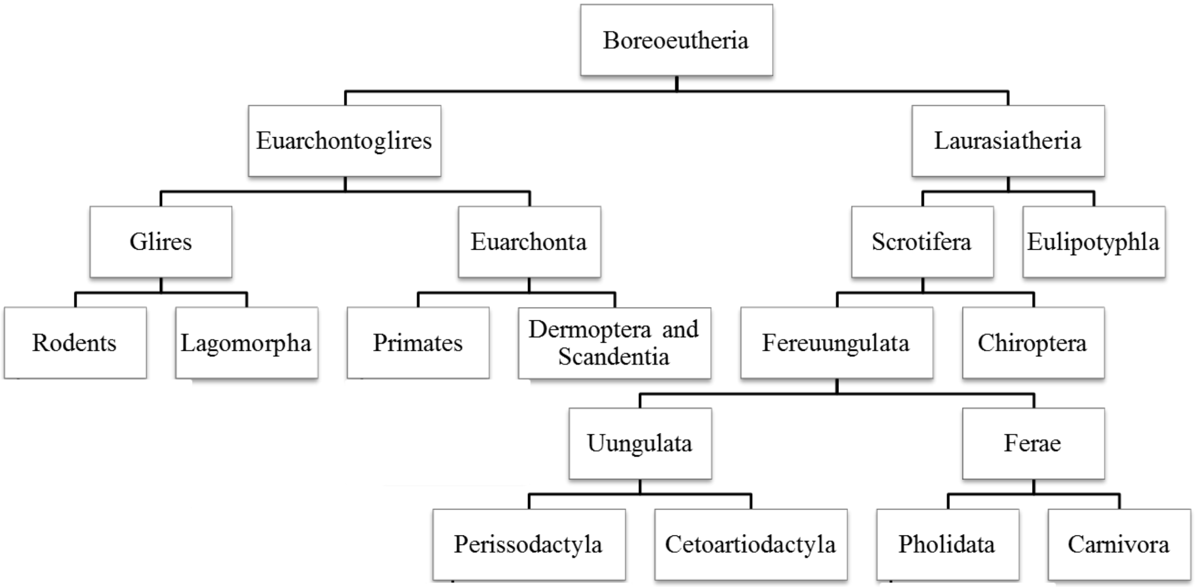

Sfig.2: Extent of conservation of GRO sequences among different species calculated using ConSurf server [7] along with the sequence diagram created by the WebLogo program [8]. The positively selected residues are marked with red bars.

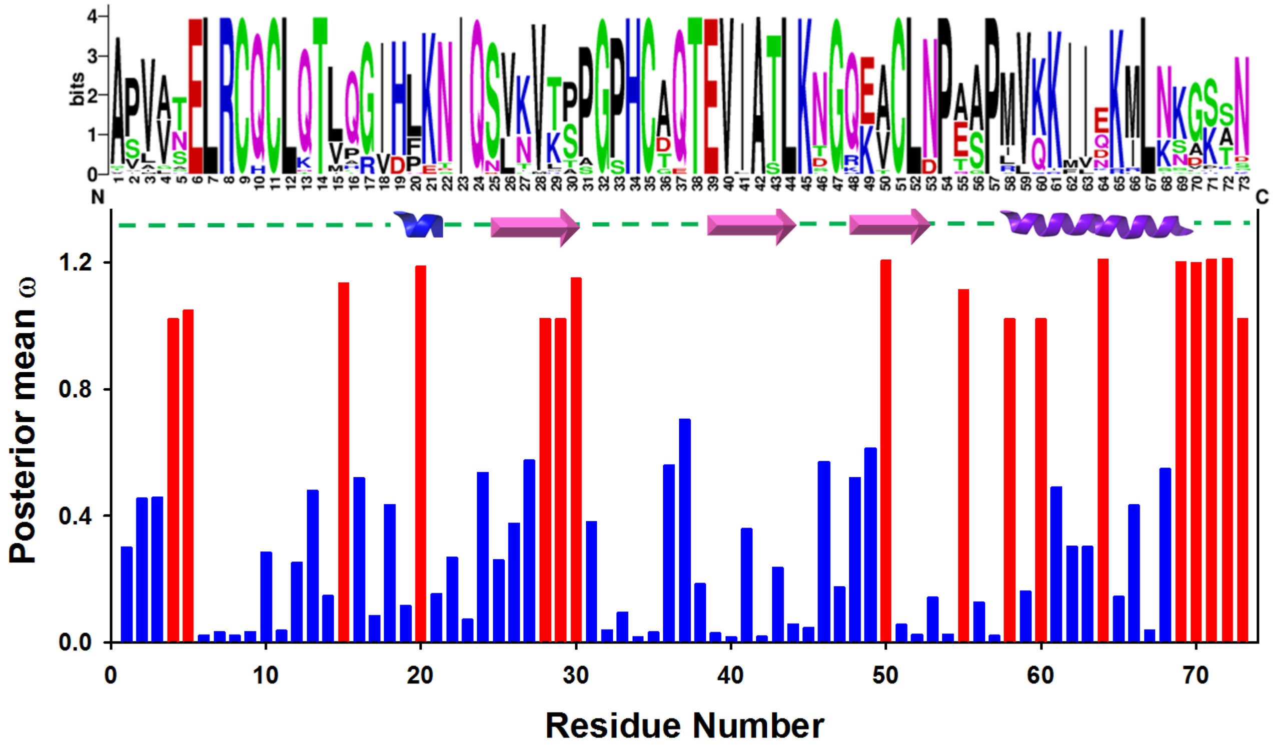

Sfig.3: Structural models for (A) MCXCL1 (NMR Model), and (B) MCXCL3, (C) HCXCL3, (D) SCXCL1, (E) SCXCL2, (F) SCXCL3, using homology modeling**.**

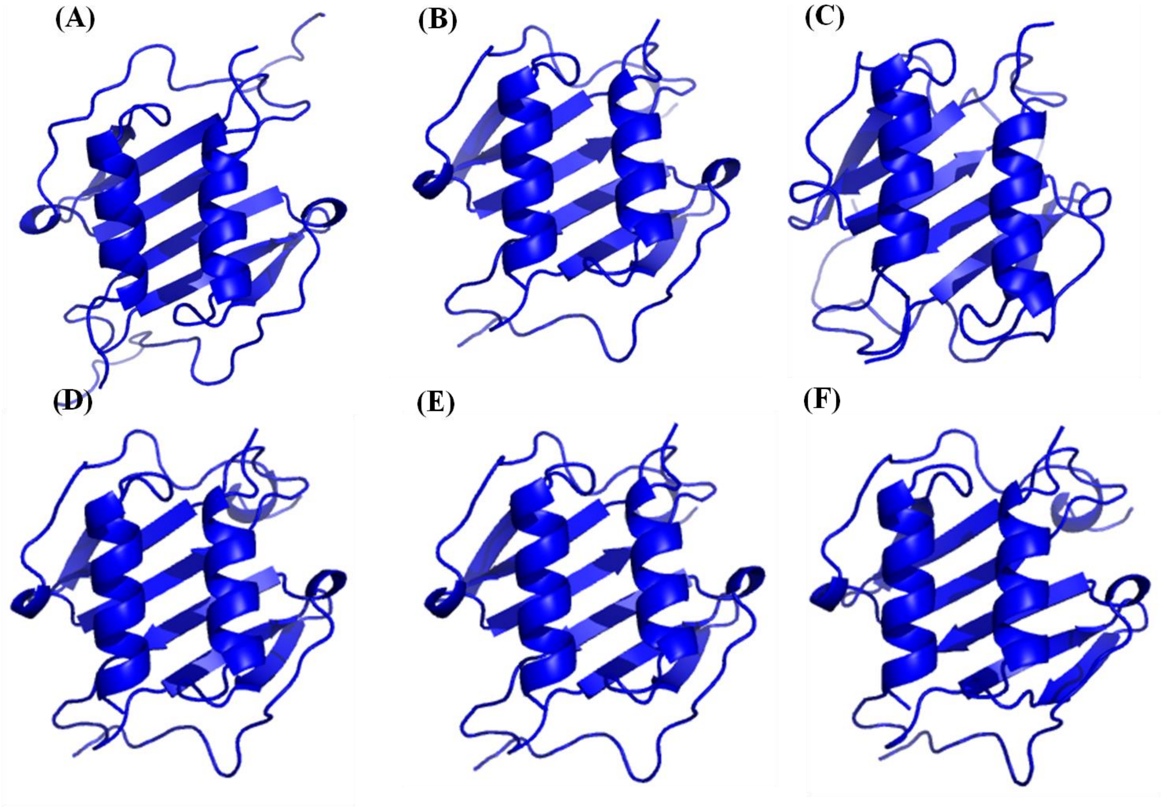

Sfig.4: Electrostatic surface potential maps for murine GRO proteins in monomeric form. The vacuum electrostatics was generated using PYMOL molecular graphics system [9].

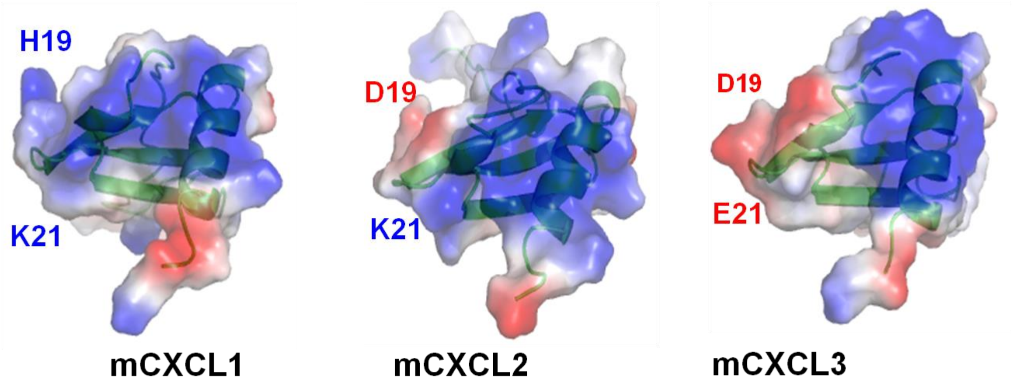

References

1. Fairbrother WJ, Reilly D, Colby TJ, Hesselgesser J, Horuk R.1994 The solution structure of melanoma growth stimulating activity. *J Mol.Biol.*242, 252-270.

2. Kim KS, Clark-Lewis I, Sykes BD.1994 Solution structure of GRO/melanoma growth stimulatory activity determined by 1H NMR spectroscopy. *J Biol.Chem.*269, 32909-32915.

3. Qian YQ, Johanson KO, McDevitt P.1999 Nuclear magnetic resonance solution structure of truncated human GRObeta [5-73] and its structural comparison with CXC chemokine family members GROalpha and IL-8. *J Mol.Biol.*294, 1065-1072.

4. Poluri KM, Joseph PR, Sawant KV, Rajarathnam K.2013 Molecular basis of glycosaminoglycan heparin binding to the chemokine CXCL1 dimer. *J.Biol.Chem.*288, 25143-25153.

5. Shao W, Jerva LF, West J, Lolis E, Schweitzer BI.1998 Solution structure of murine macrophage inflammatory protein-2. *Biochemistry*.37, 8303-8313.

6. Rajasekaran D, Keeler C, Syed MA, Jones MC, Harrison JK, Wu D, Bhandari V, Hodsdon ME, Lolis EJ.2012 A model of GAG/MIP-2/CXCR2 interfaces and its functional effects. *Biochemistry*.51, 5642-5654.

7. Ashkenazy H, Abadi S, Martz E, Chay O, Mayrose I, Pupko T, Ben-Tal N.2016 ConSurf 2016: an improved methodology to estimate and visualize evolutionary conservation in macromolecules. *Nucleic Acids Res.*44, W344-W350.

8. Crooks GE, Hon G, Chandonia JM, Brenner SE.2004 WebLogo: a sequence logo generator. *Genome Res.*14, 1188-1190.

9. Anonymous, The PyMOL Molecular Graphics System, Version 1.4.1 Schrödinger, LLC, in: Anonymous.
